# Prevalence of mental disorders in defendants at criminal court

**DOI:** 10.1192/bjo.2022.63

**Published:** 2022-05-12

**Authors:** Penelope Brown, Ioannis Bakolis, Elizabeth Appiah-Kusi, Nicholas Hallett, Matthew Hotopf, Nigel Blackwood

**Affiliations:** Department of Psychological Medicine, Institute of Psychiatry, Psychology and Neuroscience, King's College London, UK; and South London and Maudsley NHS Foundation Trust, UK; Department of Biostatistics and Health Informatics, Institute of Psychiatry, Psychology and Neuroscience, King's College London, UK; Department of Psychology, Institute of Psychiatry, Psychology and Neuroscience, King's College London, UK; Essex Partnership University NHS Foundation Trust, UK; Department of Psychological Medicine, Institute of Psychiatry, Psychology and Neuroscience, King's College London, UK; South London and Maudsley NHS Foundation Trust, UK; and Department of Forensic and Neurodevelopmental Sciences, Institute of Psychiatry, Psychology and Neuroscience, King's College London, UK

**Keywords:** Developmental disorders, forensic mental health services, human rights, psychotic disorders, psychiatry and law

## Abstract

**Background:**

Psychiatric morbidity in prisons and police custody is well established, but little is known about individuals attending criminal court. There is international concern that vulnerable defendants are not identified, undermining their right to a fair trial.

**Aims:**

To explore the prevalence of a wide range of mental disorders in criminal defendants and estimate the proportion likely to be unfit to plead.

**Method:**

We employed two-stage screening methodology to estimate the prevalence of mental illness, neurodevelopmental disorders and unfitness to plead, in 3322 criminal defendants in South London. Sampling was stratified according to whether defendants attended court from the community or custody. Face-to-face interviews, using diagnostic instruments and assessments of fitness to plead, were administered (*n* = 503). Post-stratification probability weighting provided estimates of the overall prevalence of mental disorders and unfitness to plead.

**Results:**

Mental disorder was more common in those attending court from custody, with 48.5% having at least one psychiatric diagnosis compared with 20.3% from the community. Suicidality was frequently reported (weighted prevalence 71.2%; 95% CI 64.2–77.3). Only 16.7% of participants from custody and 4.6% from the community were referred to the liaison and diversion team; 2.1% (1.1–4.0) of defendants were estimated to be unfit to plead, with a further 3.2% (1.9–5.3) deemed ‘borderline unfit’.

**Conclusions:**

The prevalence of mental illness and neurodevelopmental disorders in defendants is high. Many are at risk of being unfit to plead and require additional support at court, yet are not identified by existing services. Our evidence challenges policy makers and healthcare providers to ensure that vulnerable defendants are adequately supported at court.

Mental disorders are endemic in individuals managed by the criminal justice system (CJS) and contribute to increased all-cause mortality, risk behaviours and stigma.^1^ Early identification and intervention is a public health concern.^2^ Numerous studies have examined the prevalence of mental disorders in prisoners and, to a lesser extent, police detainees,^1,3^ but these do not include the large proportion of individuals charged with offences who are not incarcerated. Decisions on whether to continue prosecution and appropriate sentencing rely heavily on whether the defendant has particular mental health needs, yet there is a dearth of research-quality data informing these.^4^ Many criminal defendants have had no prior contact with mental health services, and high levels of morbidity go undetected and untreated.^2,5^ Identifying those who require assessment and treatment remains problematic.^6^ Since the 1990s, there has been an international drive to divert those with mental disorders away from the CJS, into community or in-patient care.^7^ Psychiatric liaison and diversion (L&D) services were established to identify vulnerable people entering the CJS at an early stage, including at court, and direct them to appropriate care pathways, with some variability across and within jurisdictions.^8,9^ These services rely heavily on referrals to identify those in need of assessment, and the true level of psychiatric morbidity cannot be determined by evaluating L&D services alone.^10^

## Mental disorder and unfitness to plead/incompetence to proceed

Particular tensions arise when defendants lack the abilities to participate effectively in their criminal trials.^11^ There is a major gap in our understanding of how mental disorder affects not only the defendant's health and risk profiles, but also their capacity to conduct and defend themselves properly in the courtroom. Fitness to plead, also referred to as fitness or competence to stand trial, refers to a defendant's ability to understand and participate in the legal processes within a criminal trial. There is international concern that large numbers of vulnerable defendants are not identified and are unfairly facing trials that they cannot fully participate in.^12,13^ The use of virtual or video hearings during the COVID-19 pandemic has raised particular concerns around procedural fairness for vulnerable defendants.^14^ In England and Wales, all criminal cases pass through the Magistrates’ Court, with more serious offences heard in the higher Crown Court. A total of 4% of such defendants are remanded into custody for further consideration at a later date, and 10% receive a prison sentence; the remainder receive acquittals, suspended or community sentences, or fines.^15^ Little is known about the prevalence of mental disorders and unmet needs in this group. In 2016, the Law Commission of England and Wales recommended significant reform of fitness to plead procedures, including screening by L&D teams, and statutory provision of intermediaries to assist defendants at trial.^13^

## Aims of the study

We have launched a study to analyse the proposed reforms and address the research gaps by exploring the relationship between mental disorder and fitness to plead.^16^ In this paper, we describe a cross-sectional study of individuals accused of criminal offences at court, which aims to determine the prevalence of mental disorders in adult defendants and to estimate the proportion likely to be unfit to plead.

## Method

### Study population

We employed two-stage sampling of 3322 criminal defendants at Camberwell Green and Croydon Magistrates’ Courts, for whom L&D services provision was provided by South London and Maudsley NHS Foundation Trust and which serve a population of 1.2 million people from the London Boroughs of Southwark and Lambeth, and Croydon and Sutton, respectively. These are among the most diverse and densely populated areas in the UK (see Supplementary Appendix available at https://doi.org/10.1192/bjo.2022.63).^17^ Around 50 adult defendants attend each court daily, with approximately 80% coming directly from the community (having been released on bail after initial arrest) and 20% from custody (either brought directly from police custody within 24 h of arrest or from remand prison), and include cases subsequently referred to the Crown Court. Court lists detailing the name, gender, date of birth and criminal charges of defendants due to attend court are published daily. An additional list of defendants attending from police custody is compiled during the day.

### Design

Sampling took place on 80 non-consecutive days between February 2015 and October 2017. The study population comprised all adult defendants on the court list (including the overnight custody list) and attending court in person (not via video link). Previous research shows a higher frequency of mental illness in defendants attending court from custody than from the community,^18^ therefore stratified random sampling was employed to oversample from the custody population (see Supplementary Appendix).

Selected defendants were checked for eligibility by the researchers (aged ≥18 years and able to communicate sufficiently in English for the purposes of the study). Defendants were excluded if they required an interpreter, displayed potentially violent behaviour/other risks or lacked capacity to consent to take part in the study and no consultee was available. Eligible defendants from the community were approached directly by the researchers. Those in custody were approached by security staff to check for risks and consent to being approached. Written consent was obtained from all participants. See Supplementary Appendix for further details about consent.

Non-identifiable demographic data (age, gender, nature of alleged offence) were collected on non-participants (including those not selected for the study and those selected but who refused or were excluded), to test the extent to which the study sample was representative of the study population.

The authors assert that all procedures contributing to this work comply with the ethical standards of the relevant national and institutional committees on human experimentation and with the Helsinki Declaration of 1975, as revised in 2008. All procedures involving human patients were approved by the National Research Ethics Service Committee London-South East (approval number 14/LO/1377), the National Offender Management Service National Research Committee (approval number 2014–225), HM Courts and Tribunal Service Data Access Panel (Privileged Access Agreement date: 10 December 2014) and the South London and Maudsley NHS Foundation Trust and King's College London Research and Development Approval (approval number R&D2015/008). Further details of obtaining ethics approval are available.^19^

### Procedures

#### Stage 1

Sociodemographic, clinical and offence-related data were collected with a study proforma, and four short screening measures for mental and neurodevelopmental disorders and unfitness to plead were administered: Prison Screening Questionnaire,^20^ Learning Disability Screening Questionnaire,^21^ Adult Attention Deficit Hyperactivity Disorder Self-Report Scale screen (ASRS version 1.1.)^22^ and a screener for unfitness to plead, derived from the Fitness-to-Plead Assessment scale (FTPA)^16^ (see Supplementary Appendix for details). All questionnaires were read to participants to ensure items were understood. Participants were categorised as screening positive if they achieved a score equal to or greater than the recommended cut-off for ‘caseness’ on one or more of the screening measures (Supplementary Appendix). Participants showing characteristics of mental disorders or reporting immediate risk of harm to themselves or others that required immediate clinical assessment were referred to the court L&D team, the prison in-reach mental health team or other services, as appropriate. All of the participants who screened positive and 15% of those who screened negative were asked to complete stage 2.

#### Stage 2

A clinical interview incorporating standardised diagnostic questionnaires was administered to stage 2 participants (immediately after or within 2 weeks of the court hearing at which they were recruited into the study): the Ammons Quick Test,^23^ to estimate IQ;^5^ MINI International Neuropsychiatric Interview (MINI) version 6.0^24^ (including the attention-deficit hyperactivity disorder (ADHD) module for childhood and current ADHD); Structured Clinical Interview for DSM-IV Axis II borderline personality disorder scale;^25^ Ritvo Autism Asperger Diagnostic Scale-Revised;^26^ Brief Psychiatric Rating Scale^27^ and FTPA.^16^ Clinical judgements as to whether participants were ‘fit to plead’, ‘borderline unfit to plead’ or ‘likely to be found unfit to plead’ were made by a forensic psychiatrist following administration of the FTPA.

### Statistical analysis

We estimated that 514 participants will provide us with a prevalence of 14% at 5% level of significance, with an allowable error of 3% (Supplementary Appendix). The sample size calculation used published data of the prison population that had a major mental disorder.^1^ Parametric (one-way ANOVA) and non-parametric tests (*χ*²-test) were employed to test differences in participants versus non-participants, selected versus non-selected screen-negative controls, participants in custody versus the community, and associations between study variables and outcomes (diagnosed mental disorder). Lifetime and current prevalence of mental disorders in defendants were estimated, taking into account the proportion of participants interviewed and testing positive for mental disorder at each stage.^28^ Post-stratification probability weighting was used to adjust for strata-specific selection probability, according to variables available for participants and non-participants.^29^ Further inverse probability weighting was carried out to adjust for differential sampling from the court and community groups, to estimate the overall prevalence of mental disorders in all court defendants. Intellectual disability was defined as an IQ of <66 combined with limited educational achievement (i.e. not higher than GCSE or equivalent), which incorporates both borderline and mild intellectual disability, as recommended by previous research (see Supplementary Appendix).^30^ All data were analysed with Stata (version 13 for Windows).

## Results

**Fig. 1:**
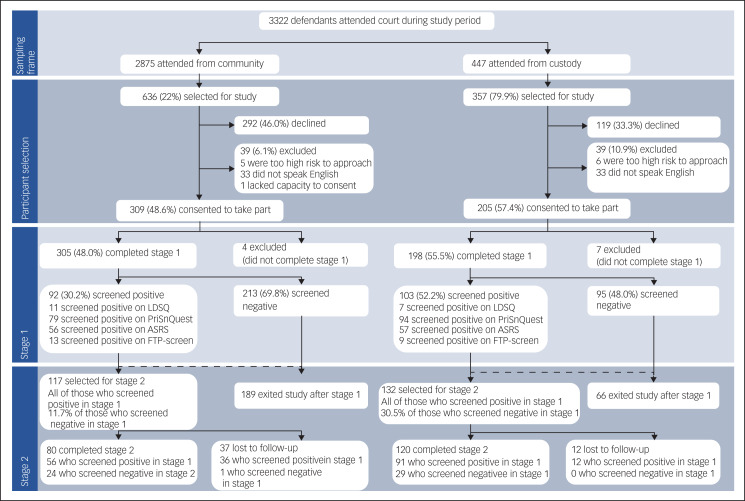
Study flowchart. ASRS, Adult Attention Deficit Hyperactivity Disorder Self-Report Scale; FTP-screen, screener for unfitness to plead, derived from the Fitness-to-Plead Assessment; LDSQ, Learning Disability Screening Questionniare; PriSnQuest, Prison Screening Questionnaire.

Figure 1 shows the study design and drop-out rates of participants. Of the 3322 defendants attending the courts during the study period, 993 were selected, 514 agreed to take part in the study and 503 completed stage 1; 9.7% of all participants were lost to follow-up. There was no evidence of age difference between participants and non-participants (*P* = 0.087), but participants were more likely to be male (87.1 *v*. 82.7%, *P* = 0.015) and charged with violent offences (45.9% of participants *v*. 35.3% of non-participants, *P* < 0.001).

Table 1 shows the demographics of the study sample, stratified by whether participants attended court from custody or the community. Participants attending from custody were more likely to be unemployed, lacking educational qualifications, come from White ethnic backgrounds and have had more convictions and court appearances than those attending from the community. Participants in custody were also more likely to have had prior contact with mental health services and be referred to the L&D team than those attending from the community.

**Table 1:** Participant characteristics (*n* = 503)

	Custody (*n* = 198), *n* (%)	Community (*n* = 305), *n* (%)	*P*-value
Gender			0.873
Men	183 (87.4)	265 (86.9)	
Women	25 (12.6)	40 (13.1)	
Age, years			0.160
Mean (s.d.), range	34.0 (11.9), 18–75	34.4 (13.1), 18–75	
Age group, years			0.304
18–25	53 (26.8)	100 (32.8)	
26–38	78 (39.4)	104 (34.1)	
39–75	67 (33.8)	101 (33.1)	
Ethnicity			0.039
White British	88 (44.4)	109 (35.7)	
White other	17 (8.6)	20 (6.6)	
Black British	41 (20.7)	72 (23.6)	
Black other	43 (21.7)	69 (22.6)	
Asian	9 (4.6)	35 (11.5)	
First language			0.210
English	173 (87.4)	254 (83.3)	
Other	25 (12.6)	51 (16.7)	
Relationship status			0.744
Single/divorced/widowed	160 (80.8)	250 (82.0)	
Married/cohabiting/civil partnership	38 (19.2)	55 (18.3)	
Employment status			<0.001
Employed/self-employed/studying	75 (37.9)	165 (54.1)	
Unemployed	123 (62.1)	140 (45.9)	
Educational attainment			<0.001
No qualification	93 (47.0)	78 (25.6)	
Up to GCSE level	80 (40.4)	135 (44.3)	
Advanced level, degree or above	26 (12.6)	92 (32.2)	
Previous mental health contact			<0.001
None	62 (32.0)	172 (56.8)	
General practitioner/primary care psychology	56 (28.9)	75 (24.8)	
Secondary mental healthcare	76 (39.2)	56 (18.5)	
Previous mental disorder			0.045
None	67 (33.8)	172 (56.4)	
Neurodevelopmental disorder (including intellectual disability)	40 (20.2)	49 (16.1)	
Anxiety/depression	93 (47.0)	119 (39.0)	
Psychosis/bipolar	51 (25.8)	36 (11.8)	
Addictions	48 (24.2)	34 (11.1)	
Personality disorder and other diagnoses	52 (26.3)	44 (14.4)	
Previous psychiatric admission			0.002
None	156 (78.8)	271 (88.9)	
At least one admission	42 (21.2)	34 (11.2)	
Current medication			<0.0001
Antidepressant/anxiolytic	31 (15.7)	37 (12.1)	
Antipsychotic	22 (11.1)	9 (3.0)	
ADHD or addictions medication	5 (2.7)	6 (1.9)	
Physical health medication	8 (4.0)	33 (10.8)	
None	132 (66.7)	220 (72.1)	
Referred to liaison and diversion team	33 (16.7)	14 (4.6)	<0.0001
Current charge			0.285
Violent offence	96 (48.73)	132 (43.85)	
Other offence	101 (51.3)	169 (56.2)	
Age at first offence, years			<0.0001
Mean (s.d.), range	19.8 (9.9), 10–67	24.9 (13.0), 8–69	
Number of previous convictions			<0.0001
None	25 (12.9)	109 (36.3)	
1–4	56 (28.9)	105 (35.0)	
5–15	45 (23.2)	55 (18.3)	
≥16	68 (35.1)	31 (10.3)	
Previous court appearances			<0.0001
None	12 (6.1)	64 (21.1)	
1–3	38 (19.2)	108 (35.6)	
4–9	39 (19.7)	67 (22.1)	
≥10	109 (55.1)	64 (21.1)	

GCSE, General Certificate of Secondary Education (standard-level school examinations taken at age 15–16 years); ADHD, attention-deficit hyperactivity disorder.

Table 2 shows the frequency of defendants who screened positive for each test in stage 1. Defendants attending court from custody were more likely to screen positive overall, including for mental illness and ADHD, than those attending from the community. To test for bias in the non-randomly selected participants who screened negative and completed stage 2, we compared those who agreed to further testing with those who were not offered (because of a lack of time or facilities) or did not agree to further testing, and detected no differences in gender (*P* = 0.340), age (*P* = 0.716), offence type (*P* = 0.243), educational achievement (*P* = 0.255), employment status (*P* = 0.903) or previous contact with mental health services (*P* = 0.130), between the subgroups.

**Table 2:** Screening test (stage 1) results by court location

Screening test	Custody (*n* = 198), *n* (%)	Community (*n* = 305), *n* (%)	*P*-value (custody versus community)
Intellectual disability (LDSQ)	7 (3.5)	11 (3.6)	0.966
Mental illness not including ADHD (PriSnQuest)	94 (47.5)	79 (25.9)	<0.0001
ADHD (ASRS)	57 (28.9)	56 (18.4)	0.006
Fitness to plead (FTP-screen)	9 (4.6)	13 (4.3)	0.879
Total screening positive	103 (52.0)	92 (30.2)	<0.0001

LDSQ, Learning Disability Screening Questionnaire; PriSnQuest, Prison Screening Questionnaire; ASRS, Attention-Deficit Hyperactivity Disorder Self-Report Scale; FTP-screen, fitness to plead screening questionnaire.

Table 3 shows the lifetime and current prevalence of psychiatric diagnoses and an estimated prevalence of those who would be found unfit or borderline unfit to plead, based on the interviewed sample, in those attending the study courts from the community and from custody. An estimation of the total prevalence of mental disorders and unfitness to plead among all defendants (adjusted for differential sampling between the court and community groups) is also shown. Half of all defendants had a lifetime history of depression, almost two-thirds were found to have childhood ADHD and just under a third had a lifetime history of psychosis. Of the current mental disorders tested for, ADHD was the most prevalent, followed by depression, borderline personality disorder, anxiety and psychosis. The prevalence of all mental disorders was notably higher in defendants attending court from custody than in the community sample.

**Table 3:** Weighted lifetime and current prevalence of mental disorders and fitness to plead in study population

	Custody sample (*n* = 198)		Community sample (*n* = 305)		Weighted total^a^ (*n* = 503)	
	%	95% CI	%	95% CI	%	95% CI
Psychotic symptoms (lifetime)	31.9	23.8–41.3	24.6	16.0–35.9	29.5	23.0–36.9
Psychotic symptoms (current)	12.0	8.0–17.5	4.5	2.7–7.6	8.4	6.1–11.6
Mania (lifetime)	10.1	5.7–17.2	8.1	3.8–16.3	9.9	5.4–16.0
Mania (current)	1.4	0.4–4.3	0.7	0.2–2.8	0.94	0.35–2.5
Depression (lifetime)	50.5	41.4–59.6	46.7	35.8–58.0	47.4	40.0–54.9
Depression (current)	21.9	16.6–28.4	7.4	5.0–11.0	14.3	11.2–18.0
Anxiety (current)	13.0	8.9–18.6	5.4	3.4–8.5	9.0	6.6–12.2
PTSD (current)	9.9	6.4–14.9	3.9	2.2–6.8	6.2	4.3–9.0
Intellectual disability	5.6	3.1–9.9	1.8	0.8–3.9	2.5	1.1–5.7
Autistic spectrum disorder	10.5	6.8–15.7	5.3	3.2–8.5	8.4	6.1–11.5
ADHD (adult)	25.0	19.3–31.7	6.0	3.8–9.4	16.4	13.2–20.3
ADHD (child)	68.0	58.6–76.1	48.8	37.0–60.8	64.5	57.0–71.4
Borderline personality disorder	19.6	14.4–26.0	5.2	3.2–8.4	13.1	10.2–16.9
Current suicidality	77.7	68.8–84.6	59.1	46.9–70.4	71.2	64.2–77.3
Unfit to plead	3.3	1.5–7.1	1.8	0.8–3.9	2.1	1.1–4.0
Borderline fit to plead	3.9	1.9–7.7	1.8	0.8–4.0	3.2	1.9–5.3

For depression and anxiety, ‘current’ refers to symptoms occurring in the previous 2 weeks and 6 months respectively. For all other diagnoses, ‘current’ refers to symptoms occurring in the previous month. PTSD, post-traumatic stress disorder; ADHD, attention-deficit hyperactivity disorder.

a Study population estimate adjusted by sampling weight (inverse of sampling fraction for each strata).

Table 4 shows the frequency of current psychiatric diagnoses per individual participant in the interviewed sample (excluding the lifetime diagnoses, suicidality and fitness to plead variables shown in Table 3). Just under half of the defendants attending court from custody and one in five attending from the community were found to have at least one current psychiatric diagnosis. Of these 158 individuals, 37 (23.4%) had received no previous mental health treatment (including from their general practitioner) and 129 (81.6%) were not referred to L&D services (79.2% of those from custody and 85.5% of those from the community). Conversely, of those with no mental disorder identified in the study, 18 (5.2%) were referred to court L&D services (12.8% of those from custody and 2.1% from the community). In addition, two individuals from custody were found to have dementia, although this was not a disorder included in the diagnostic questionnaires.

**Table 4:** Frequency of psychiatric diagnoses per participant

Number of current psychiatric diagnoses per participant	Custody (*n* = 198), *n* (%)	Community (*n* = 305), *n* (%)
0	102 (51.5)	243 (79.7)
1	26 (13.1)	24 (7.9)
2	17 (8.6)	15 (4.9)
3	17 (8.6)	9 (3.0)
≥4	36 (18.1)	14 (4.6)

## Discussion

At a time when there are concerns that the CJS is failed by mental health services,^31^ this study highlights the extent of psychiatric morbidity in criminal defendants. In recent years, the UK has seen the rollout of court L&D schemes alongside new guidance considering the prosecution and sentencing of defendants with mental disorder, yet robust data on mental health in this population has been lacking.^4^ This is the first study to examine the prevalence of a wide range of mental disorders in defendants attending criminal court in the UK. Until now, policy makers have relied on a 1999 study carried out by Shaw et al,^18^ which estimated levels of serious mental illness in Magistrates’ Court defendants aged 21–38 years.

### Main study findings: prevalence of mental disorders

We found a high burden of mental disorders in the criminal defendant population, with one in two defendants from custody and one in five from the community reporting symptoms of at least one psychiatric condition. Levels of common affective disorders in our sample are slightly higher than those found in the general population, according to the 2014 Adult Psychiatric Morbidity Survey (APMS), which found a national 43.4% lifetime prevalence and 15.7% point prevalence of anxiety and depression (18% point prevalence in London).^32^ The prevalence of intellectual disability estimated in defendants in our study (2.5%) is marginally higher than in the general adult population (2.16%).^33^ Of particular concern is our finding that symptoms of major mental illness, neurodevelopmental disorder (autism spectrum disorder (ASD) and ADHD) and borderline personality disorder were notably higher in our study of court defendants compared with the APMS (which found a prevalence of 4.4% for post-traumatic stress disorder, 0.7% for psychosis, 0.8% for ASD, 9.7% for ADHD and 2.4% for borderline personality disorder). Our estimates are comparable to the figures found in police detainees, including in a recent study using similar methodology in the South London population that serves the courts in our study (which found a prevalence of 22.4% for depression, 6.7% for psychosis, 8.2% for post-traumatic stress disorder and 11.2% for ADHD).^3^ Suicidal thoughts were reported in over 70% of court defendants, which is significant when compared with the one in 20 reported for the general population in the APMS and one in five for police detainees.^3^

As in the UK, the international data on mental health in the defendant population is limited. One Canadian study used linked health and justice administrative data to explore the prevalence of mental disorders in all those involved in the justice system.^6^ This found similarly elevated levels of mental disorders among individuals accused of crimes when compared with the general population, with 38.9% found to have any major mental disorder.^6^ A 2009 study in Australian Magistrates’ Courts (*n* = 60) found that 38% of those interviewed had a mental health problem, with 10% estimated as having an IQ <70, 33% reporting affective/neurotic disorders and 10% reporting a psychotic disorder.^34^ Our findings support and extend the work carried out by Shaw et al, which also reported substantial levels of depression and psychosis in criminal defendants, in particular those attending court from custody (6.6% compared with 1.3% in the community sample), with only a small proportion referred to L&D services.^18^ Post-traumatic stress disorder has also been found to be overrepresented in offender populations, and is associated with numerous comorbidities, including substance misuse and suicidality.^35^ It is recognised that both prison and court can be re-traumatising for individuals who have themselves been victims of trauma, and the recommendations for introducing trauma-informed approaches in prisons should equally apply to court proceedings.^36^ An earlier study carried out in North England found 38.8% of court defendants coming from police custody were thought to have a mental disorder, including substance misuse.^37^ Of 136 defendants, only one was found to have psychosis, with an additional defendant suffering from chronic psychosis and drug misuse. Only three were diagnosed with depression. This study used clinical judgement rather than structured diagnostic assessments to identify mental disorders, which could partly account for lower levels of mental disorders, as well as different sociodemographic data between North England and London. Nevertheless, the study highlighted high levels of vulnerability and deprivation, and the importance of identifying and addressing the health and other needs of criminal defendants has long been recognised.

### Neurodevelopmental disorders in the CJS

In recent years there has been increasing interest in neurodevelopmental disorders (ASD, ADHD and intellectual disability) in the CJS.^38,39^ Although there is little consensus on how these disorders affect offending behaviour, it is widely accepted that individuals with neurodevelopmental disorders experience difficulties at all stages of the CJS, including at court, where communication difficulties, maladaptive coping strategies, suggestibility and the risk of false confessions are particular concerns.^40,41^ Our results suggest a slight overrepresentation of intellectual disability in court defendants compared with the general population, but the figure falls below previous estimates suggesting 5–10% of adult offenders have intellectual disability.^30,42^ This could be a result of early diversion from the CJS or methodological issues, including small sample size and poor validity of IQ estimates in individuals for whom English is not their first language, a group who are overrepresented in the study population (national comparator 8%).^17^ We estimate that around 16% all defendants have ADHD in adulthood. This is in keeping with research in prisoners estimating that one in four adult prisoners have ADHD.^39^ Our estimation that up to one in ten defendants have ASD is also in keeping with literature suggesting that ASD is overrepresented in the CJS, although the research in this area is mixed and there is no clear consensus about the prevalence of ASD in offenders.^38^ Our findings are significant as not only are these disorders overrepresented in court defendants, but many are going unrecognised, yet likely to significantly affect an individual's ability to take part in their trial, i.e. their fitness to plead.

### Unfitness to plead

When concerns are raised about a defendant's fitness to plead, expert psychiatric evidence is required.^11^ A final determination of unfitness to plead can only be made by a judge on the basis of psychiatric evidence, which introduces additional costs and long delays in the CJS. For those found unfit to plead, hospital or community-based supervision and treatment orders are often made.^43^ Although this study does not seek to establish the prevalence of findings of unfitness to plead, we are able to estimate the proportion in whom unfitness to plead is clinically suspected. We used clinical judgement in combination with the FTPA, a standardised assessment instrument, to consider whether participants met the legal criteria for fitness to plead, including the foundational abilities required to effectively participate in court and the associated decision-making abilities.^16^ Overall, we estimated that 2.1% of defendants would be unfit to plead if screening were to be implemented, and a further 3.2% were deemed to have borderline understanding and abilities. This has significant resource implications. In 2018, 1.44 million defendants were directed to appear at Magistrates’ Courts in England and Wales. Extrapolation of our results yields an estimated annual rate of 30 240 defendants being found unfit to plead and just over 46 000 more requiring psychiatric assessment to determine whether they can participate effectively in their trials. Although many ‘borderline unfit’ defendants may manage with additional support at court, such as registered intermediaries, courtroom adjustments or delayed trials, a proportion are likely to remain unable to participate effectively at trial regardless of the level of support provided for them.^13^ Currently, on average, 100 individuals are found unfit to plead in England and Wales each year.^43^

The Law Commission has highlighted the lack of data in relation to vulnerable defendants at court, which has made it difficult to estimate the resource implications of their proposals,^13^ yet incorporation of their recommendations into policy could vastly increase the number of defendants assessed and found unfit. This, in turn, will have significant resource implications for the justice system and add pressure to already overstretched mental health providers. They estimate that, in addition to the 100 defendants currently found unfit, a further 60 defendants at Crown Court and 800 at Magistrates’ Court are likely to lack the abilities needed to participate effectively at trial if their recommendations are implemented. Our findings suggest that their prediction that only 960 out of 1.44 million (0.07%) of criminal defendants would be unfit to plead is a gross underestimation, especially if screening for mental disorder and unfitness to plead becomes routine.

### Strengths and limitations

There are numerous obstacles to high-quality empirical research in the CJS,^19^ yet despite these challenges, we were able to carry out a methodologically robust study on a large and diverse cohort of criminal defendants. Around half of those invited to take part agreed to be interviewed. A particular strength of this study was the use of structured clinical assessments validated in offender populations^21,44^ and conducted by trained clinicians on defendants as they attended court. We used similar methodology to the study by Shaw et al, but went further by exploring mental illness, personality disorder, neurodevelopmental disorders and unfitness to plead in all adult defendants at Magistrates’ Court, including those charged with serious offences who were subsequently referred to Crown Court.

Although adequately powered, the number included in the final sample was limited by constraints in access to the custody area and interview rooms, and sensitive working around court procedures and the experiences of the defendant. This restricted the use of non-response rates and sampling weights in the analysis, as well as limiting meaningful analysis of the prevalence of mental disorders by other measures of vulnerability, such as gender and ethnicity. Approximately 20% of the screened population were lost to follow-up and did not complete phase 2. Reasons for non-participation and drop-out could not be explored, but there is evidence that non-participation is associated with poorer mental health, which may therefore be associated with underestimation of psychiatric morbidity in prevalence studies.^45^ In addition to participation bias, the study methodology is also inherently prone to reporting bias. There are particular limitations in using the MINI, which has been found to over-diagnose certain mental disorders in offender populations, especially when administered at a highly stressful period (such as attending court).^44^

A limitation of the study is the lack of generalisability. Although the study was carried out at two sites serving demographically different populations, both courts are based in densely populated and ethnically diverse areas of South London. Therefore, our findings may not be representative of the population as a whole, as social and economic conditions are known to directly influence the prevalence and severity of mental disorders.^46^

To summarise, we found high levels of mental illness and neurodevelopmental disorders among criminal defendants at Magistrates’ Court, with a significant proportion likely to be unfit to plead. The sociodemographic determinants for mental disorder, and the impact of symptoms on an individual's ability to participate effectively at trial requires further exploration. Although L&D services have been rolled out nationally and internationally, their effectiveness is yet to be fully evaluated, and is likely to be limited by their reliance on referrals into the service. Defendants with mental health difficulties attending from the community are at particular risk of not having their needs identified. Whether the CJS and L&D services are adequately resourced to fulfil their intended aim ‘to ensure an individual's ability to participate effectively in the criminal justice process’ remains to be seen.

## Data Availability

The data that support the findings of this study are available from the corresponding author, P.B., upon reasonable request.

## Supplementary material

For supplementary material accompanying this paper visit http://doi.org/10.1192/bjo.2022.63. click here to view supplementary material
