# Supplementary material for: Prevalence of mental disorders in defendants at criminal court
Source: BJPsych Open. 2022 May 12;8(3):e92. doi: 10.1192/bjo.2022.63 (PMC9169500; doi:10.1192/bjo.2022.63)
Supplement: Supplementary file 1 [file S2056472422000631sup001.docx]

Supplementary Appendix to “Prevalence of mental disorder in defendants at criminal court “

**Appendix A: Diversity of study population**

The London boroughs of Southwark, Lambeth, Croydon and Sutton have 10-30 times the national average population density. In Sutton, 23.1% of the population are from black, Asian and minority groups, with this figure rising to 43-47% in the other study boroughs, with the national comparator at 14.6%. Nationally, 13.3% of residents are born abroad, and 8% do not have English as their first language. In our study population the equivalent figures are 23.1% and 10% for Sutton; 29.4% and 14.5% for Croydon; 32.2% and 20.3% for Lambeth; and 38.4% and 19.6% for Southwark. Unemployment rates in our study population range from 4.1% (Croydon) to 7.7% (Southwark), compared to a 5.1% national unemployment rate.

**Appendix B: Sampling strategy and sample size calculation**

All Magistrates court cases are heard on weekdays, with overnight custody cases also heard on Saturdays and public holidays. A forensic psychiatrist is present in the court L&D service on Tuesdays (Camberwell Green) and Wednesdays (Croydon), which can lead to more cases with concerns about mental disorder being listed on those dates. To ensure a representative study sample of the whole court population, data collection was evenly spread across the week, including Saturdays and bank holidays. Sampling took place on non-consecutive days in order to reduce the burden on the court and security staff.

Stratified random sampling was employed to oversample from the custody population to maximise the number of cases of mental disorder for meaningful analysis, using a sampling fraction 4/5 from custody compared to 1/5 for the community population. Each listed defendant was allocated a number, and a random number generator was employed within each stratum to select participants for the study. Two-stage sampling was employed which utilises a brief screen for mental disorder and unfitness to plead in all study participants, followed by a longer structured clinical interview to confirm the presence of mental disorder and fitness to plead in only those participants with high scores who screened “positive”, and a small selection of those who screened negative. This method was chosen to reduce the burden on the courts and participants by minimising the number of longer interviews, while maintaining robust methodology.

Using the formula n = z^2^P(1-P)/d^2^ where n= sample size, z= level of confidence (1.96 for 95% confidence level), P= expected prevalence (14%) (according to previous research) and d=allowable error (3%), we arrived at a suggested n=514. We recruited 514 participants into the study, of which 11 were unable to complete stage 1 due to time constraints at court. Therefore, the final sample comprised 503 participants.

**Appendix C: Consent procedures**

Eligible defendants from the community were approached directly by the researchers. Those in custody were approached by security staff to check for risks and consent to being approached. Potential participants were given written and oral information about the study, including confidentiality limitations. These were developed with input from service user groups, including individuals with experience of going through the Criminal Justice System. Eligible defendants who agreed and had capacity to consent provided written consent to take part. Participants were asked to consent to the researchers accessing their medical records and police national computer record to verify the information they provided regarding medical and offending history. They were not excluded from the study if they refused to consent to information gathering.

For those assessed as lacking capacity to consent, consultees were sought to give an independent assessment of whether they would be willing to take part, in accordance with the Mental Capacity Act 2005. Where a consultee (personal or professional) was not available, the individual was excluded from the study.

**Appendix D: Instrument and Cut-off details**

The stage 1 screening proforma (see below, Appendix E) comprised demographic and clinical information, followed by four screening tests for mental disorder/unfitness to plead: the Prison Screening Questionnaire (PriSnQuest), an eight-item instrument validated to screen for mental illness in CJS populations; the seven-item Learning Disability Screening Questionnaire (LDSQ); the six-question Adult Attention Deficit Hyperactivity Disorder (ADHD) Self Report Scale screen(ASRS-v1.1.); and a five-item screen for unfitness to plead, derived from the FTP Assessment Instrument (FTPA). All questionnaires were read to participants to ensure items were understood. Participants were rated as “screen positive” if they scored above the established cut-off on any one of the screening tests (PriSnQuest score ≥3/8, LDSQ score <46%, ASRS score ≥4/6 or FTPA score <5/5).

The stage 2 diagnostic questionnaire comprised : the Ammons Quick Test (AQT),^21^ a brief test of intellectual functioning validated to estimate IQ;^4^ an abbreviated version of the MINI International Neuropsychiatric Interview (MINI) v6.0,^22^ a structured diagnostic interview for mental disorders according to the ICD-10 and the DSM-IV (see below); the Structured Clinical Interview for DSM-IV Axis II (SCID II) borderline personality disorder;^23^ the Ritvo Autism Asperger Diagnostic Scale-Revised (RAADS-R)^24^ to identify the presence of autistic spectrum disorder (ASD); the Brief Psychiatric Rating Scale (BPRS),^25^ to evaluate severity of current symptoms of mental disorder; and a structured assessment of fitness to plead using the FTPA.^14^

The mental disorders assessed in the abbreviated MINI were:

- Major Depressive Disorder (current and lifetime)
- Suicidality (assessed as “low”, “moderate” or “high”)- participants who scored in an of these categories were included as reporting “current suicidality”
- Mania and hypomania (current and lifetime) – only those who met the threshold for “mania” were included in the analysis
- Obsessive Compulsive Disorder (current)- not included in the final analysis as not screened for in stage 1
- Post-traumatic stress disorder (current)
- Alcohol and substance dependence/abuse (current)- not included in the final analysis as not screened for in stage 1
- Psychotic disorder (current and lifetime)
- Generalised anxiety disorder (current)
- Antisocial personality disorder (lifetime)
- ADHD (childhood and current)

The AQT has been found to correlate well with the Wechsler Adult Intelligence Scale(WAIS-R) Full Scale IQ, an was used to estimate IQ and intellectual disability in this study. Lack of current norms in the Criminal Justice System for the AQT have led to concerns of overestimation of the true prevalence of intellectual disability when the usual cut-off of IQ<70 is used. Previous research has compensated for this by using a lower IQ equivalence threshold (Hassiotis *et al* (2011)- in main reference). We have replicated this methodology such that intellectual disability (ID) was defined as a score of 25 or less on the AQT (equivalent to an IQ of <66) combined with limited educational achievement (i.e. not higher than GCSE or equivalent) which incorporates both borderline and mild ID. Any participants with an AQT score ≤25 but reporting educational attainment higher than “GCSE” Level were included in the normal ability group.

**Appendix E: Stage 1 Screening Proforma**

Participant’s study ID: ⁪⁪⁪⁪ Location: Community□ Custody□ Date of assessment:⁪⁪ / ⁪⁪/⁪⁪

Assessment completed by _______________________

**PROFILE**

**Month and year of birth ……./………**

**Age last birthday** ⁪⁪ ……………..

**Gender** M/F

**Ethnicity**

1.White British ⁪ 2.Other White ⁪

3.Black Caribbean 4.Black African ⁪

5.South Asian ⁪ 6.Chinese ⁪

7.Mixed White/Black ⁪ 8.Mixed White and Asian ⁪

9.Other (please state) ......................................................

**Country of Birth ………………………………………**

**Relationship status**

1. Single 2. Divorced/dissolved civil partnership ⁪

3. Married/civil partnership ⁪ 4. Widowed ⁪

5. Cohabiting 6. Other (please specify).............................

**Living situation**

1.Alone ⁪ 2.With partner ⁪

3.Hostel/bail hostel 4.Homeless ⁪

5.Living with parents or family ⁪ 6.With others (specify)………………………....⁪

7.Other (please specify)…………………………… ⁪

⁪

**Housing Tenure**

1.Own Home 2.Private rented

3.Council rented 4.Other

**Employment status**

1.Employed full-time ⁪ 2.Employed part-time ⁪

3.Self-employed ⁪ 4.Student ⁪

5.Retired ⁪ 6.Unemployed ⁪

7.Looking after home/family ⁪ 8.Disabled/on sick leave ⁪

9.Other (please specify) ⁪ ......................................................

**Education**

Age when left school ……………………… ⁪⁪

Highest qualification:

*1. O Levels/GCSE 2. A Levels 3. Certificate*

*4. Diploma 5. Degree 6. Other…………………….*

**Mental Health History**

Have you seen a doctor about your mental health in the past? Y/N

**Existing mental health or neurodevelopmental diagnoses**

ADHD diagnosed in childhood ⁪ ADHD diagnosed as adult ⁪

ASD ⁪Learning disability ⁪

Anxiety disorder/OCD ⁪ Bipolar disorder ⁪

Depression ⁪Personality disorder ⁪

Psychotic disorder ⁪PTSD ⁪

Alcohol dependence ⁪ Substance abuse

Other (please specify) ………………………….

Previous hospital admissions………………

**Any other medical conditions (e.g. asthma, diabetes)………………………….**

**GP Details (if consented) ……………………………………………………………**

**Offending History**

**Current Charge(s) …………………………………**

**Previous Offences**

Number of previous convictions …………………………………

Age at first offence ………………………………..

**Offence type**

Burglary ⁪ Drug offences ⁪

Fraud & Forgery ⁪ Motoring offences ⁪

Robbery ⁪ Theft & Handling ⁪

Sexual offences ⁪ Violence against the person ⁪

Other offence (please specify) ⁪ _______________________________

**Have you ever attended court before?** YES / NO

i) If YES, how many times have you attended court?

1-3

4-6

7-9

10+

ii) Have you attended court as a: (please tick)

Defendant Juror

Witness Public Gallery

Barrister Expert Witness

Defendant Support Victim Support

Other (Please list…………………………………)

**How familiar are you with courtroom procedures?**

| 1 | 2 | 3 | 4 | 5 |
| --- | --- | --- | --- | --- |
| Very Unfamiliar | Somewhat Unfamiliar | Neither familiar nor unfamiliar | Somewhat Familiar | Very Familiar |

**LDSQ**

**Please ask the participant to complete one of the LDSQ sets then complete the table below by ticking or marking a cross in the appropriate box.**

| **LDSQ set used (circle) A B C D E** | Yes | No | Don’t Know |
| --- | --- | --- | --- |
| 1. Can the person tell the time?  Time given by participant__________________ | 1 | 0 | 0 |
| 2. Can the person read? | 1 | 0 | 0 |
| 3. Can the person write? | 1 | 0 | 0 |
| 4. Has the person lived independently?  (Can be with family if not dependent on them) | 1 | 0 | 0 |
| 5. Has the person previously had a job for more than 6 months? | 1 | 0 | 0 |
| 6. Has the person had previous contact with learning disability services? | 0 | 1 | 0 |
| 7. Has the person had special schooling? | 0 | 1 | 0 |
| **Total number of points** |  |  |  |
| **Answered (7 – number of ‘don’t know’ responses)**  **(If this is less than five, the score is not valid)** |  |  |  |
| **Percentage score**  7/7=100, 6/7=86, 5/7=71, 4/7=57**, 3/7=43, 2/7=29, 1/7=14.** 6/6=100, 5/6=83, 4/6=67, 3/6=50**, 2/6=33.** 5/5=100, 4/5=80, 3/5=60**, 2/5=40, 1/5=20.** |  |  |  |

**LDSQ result**

Unlikely to have LD (>46%)=LDSQ negative⁪ Likely to have LD (<46%)= LDSQ positive ⁪

⁪

**The Prison Screening Questionnaire (PriSnQuest)**

(Shaw et al, 1999)

**Please put a Cross (X) in the box (Yes, No) that best describes how you have felt in the past year.**

|  |  | **Yes** | **No** |
| --- | --- | --- | --- |
| 1 | Have you previously seen a psychiatrist? |  |  |
| 2 | Have you been taking longer over the things you do? |  |  |
| 3 | Have you recently been able to enjoy your normal every day activities? |  |  |
| 4 | Have you recently felt that life isn’t worth living? |  |  |
| 5 | Have you recently found yourself wishing you were dead and away from it all? |  |  |
| 6 | Have you recently felt that your thoughts have been directly interfered with, or controlled by another, in a way that people would find hard to believe? |  |  |
| 7 | Have there recently been times when you felt that people were plotting to cause you serious harm or injury? |  |  |
| 8 | Have you recently heard voices saying a few words or sentences when there was no one around to account for this? |  |  |

**Scoring:**

Items 1-2 & 4-8: Score 1 for each ‘Yes’, 0 for each ‘No’.

Item 3: Score 1 for ‘No’, 0 for ‘Yes’.

Score 3 or more = PrisnQuest positive

Fitness to Plead screen

(derived from Brown *et al.* (2018)

|  |  | **0/1** |
| --- | --- | --- |
| 1 | What are you charged with? |  |
| 2 | If you plead not guilty, what does this mean? |  |
| 3 | If you plead guilty, what does this mean? |  |
| 4 | What is the role of the defence lawyer? |  |
| 5 | What does evidence mean? |  |

Scoring (score <5= FTP positive)

Q1

| **1 point** | A correct response according to court listings |
| --- | --- |
| **0 points** | An incorrect or no response |

Q2

| **1 point** | A response reflecting one of the general ideas listed. | |
| --- | --- | --- |
| **0 points** | A trivial or unrelated concept. | |
| **General Concept:** | | **Examples:** |
| 1. I did it | | “I committed the crime”  “I’m guilty as charged”  “Admitting I did it” |
| 2. I’m responsible | | “taking responsibility for it”  “accepting liability/responsibility” |
| 3. Lower sentence | | “to get lenient sentence”  “to have a lighter sentence”  “thoughts of greater leniency” |
| 4. Not having a trial | | “there is not going to be a trial” |

Q3

| **1 point** | A response reflecting one of the general ideas listed. | |
| --- | --- | --- |
| **0 points** | A trivial or unrelated concept. | |
| **General Concept:** | | **Examples:** |
| 1. I didn’t do it | | “I didn’t commit the crime”  “I’m not guilty as charged”  “I didn’t do it” |
| 2. I’m not responsible | | “not taking responsibility for it”  “denying liability/responsibility”  “not being culpable” |
| 3. Prosecution can’t prove it | | “the case won’t be proved beyond reasonable doubt”  “prosecution won’t be able to prove the case”  “prosecution will have to gather evidence to prove I did it” |
| 4. Having a trial | | “there is going to be a trial” |

Q4

| **1 point** | A response reflecting one of the general ideas listed. | |
| --- | --- | --- |
| **0 points** | A trivial or unrelated concept. | |
| **General Concept:** | | **Examples:** |
| 1. To defend the client | | “to defend you”  “defends the allegations”  “to defend the client”  “tries to prove I’m not guilty” |
| 2. To examine evidence | | “present evidence to show innocence”  “to present evidence in my defence”  “to show evidence why it was not me”  “questions witnesses” |
| 3. To present case/ Follow the client’s instructions | | “to argue case for the defendant”  “represent the defendant”  “present a case favourable to the defendant” |
| 4. Mitigation | | “to show why I did what I did was not at all my fault”  “ to show why my sentence should be more lenient”  “highlight any mitigating circumstances” |
| 5. Challenge prosecution | | “to question prosecution evidence”  “to challenge the case presented by the prosecution”  “argue against what prosecution say” |
| 6. Advise the client | | “to explain court proceedings to me”  “to explain what my choices are” |

Q5

| **1 point** | A response reflecting one of the general ideas listed. | |
| --- | --- | --- |
| **0 points** | A trivial or unrelated concept. | |
| **General Concept:** | | **Examples:** |
| 1. Mentions a piece of evidence | | “DNA”  “blood”  “hair sample” |
| 2. Presented in court | | “reliable information used in the court”  “produced in courtroom to prove a point”  “presented by barristers in court” |
| 3. Makes fact more/less likely | | “used in either defence/prosecution to argue guilt/innocence”  “can link me with the crime or away from the crime”  “proof”  “things that either prove/disprove arguments presented” |
